# Polyester production by halophilic and halotolerant bacterial strains obtained from mangrove soil samples located in Northern Vietnam

**DOI:** 10.1002/mbo3.44

**Published:** 2012-10-11

**Authors:** Doan Van-Thuoc, Tran Huu-Phong, Nguyen Thi-Binh, Nguyen Thi-Tho, Duong Minh-Lam, Jorge Quillaguamán

**Affiliations:** 1 Department of Microbiology and Biotechnology, Faculty of Biology, Hanoi National University of Education136 Xuan Thuy, Cau Giay, Hanoi, Vietnam; 2 Center of Biotechnology, Faculty of Sciences and Technology, Universidad Mayor de San SimónCochabamba, Bolivia

**Keywords:** Biopolyesters, halophilic bacteria, halotolerant bacteria, mangrove forests, polyhydroxyalkanoates

## Abstract

This research article reports halophilic and halotolerant bacteria isolated from mangrove forests located in Northern Vietnam. Several of these bacteria were able to synthesize polyhydroxyalkanoates (PHAs). PHAs are polyesters stored by microorganisms under the presence of considerable amounts of a carbon source and deficiency of other essential nutrient such as nitrogen or phosphorous. Mangrove forests in Northern Vietnam are saline coastal habitats that have not been microbiologically studied. Mangrove ecosystems are, in general, rich in organic matter, but deficient in nutrients such as nitrogen and phosphorus. We have found about 100 microorganisms that have adapted to mangrove forests by accumulating PHAs. The production of polyesters might therefore be an integral part of the carbon cycle in mangrove forests. Three of the strains (ND153, ND97, and QN194) isolated from the Vietnamese forests were identified as *Bacillus* species, while other five strains (QN187, ND199, ND218, ND240, and QN271) were phylogenetically close related to the α-proteobacterium *Yangia pacifica*. These strains were found to accumulate PHAs in noticeable amounts. Polymer inclusions and chemical structure were studied by transmission electron microscopy and proton nuclear magnetic resonance (NMR) spectroscopy analyses, respectively. Strains ND153, ND97, QN194, QN187, ND240, and QN271 synthesized poly(3-hydroxybutyrate) (PHB) from glucose, whereas strains ND199 and ND218 synthesized poly(3-hydroxybutyrate-*co*-3-hydroxyvalerate) (PHBV) from this carbohydrate. With the exception of strain QN194, the strains accumulated PHBV when a combination of glucose and propionate was included in the culture medium. The polymer yields and cell growth reached by one *Bacillus* isolate, strain ND153, and one Gram-negative bacterium, strain QN271, were high and worth to be researched further. For experiments performed in shake flasks, strain ND153 reached a maximum PHBV yield of 71 wt% and a cell dry weight (CDW) of 3.6 g/L while strain QN271 attained a maximum PHB yield of 48 wt% and a CDW of 5.1 g/L. Both strain ND153 and strain QN271 may only represent a case in point that exemplifies of the potential that mangrove forests possess for the discovery of novel halophilic and halotolerant microorganisms able to synthesize different types of biopolyesters.

## Introduction

Microbial polyesters, also known as polyhydroxyalkanoates (PHAs), are synthesized by various microorganisms as a physiological strategy related to the utilization of nutritional resources in an ecosystem. When a carbon source is in excess in the ecosystem, while other essential nutrient (e.g., nitrogen, phosphorous, or oxygen) is insufficient to promote cell growth, the carbon source may be transformed to polyesters as intracellular carbon and energy storage compounds for the cells (Steinbüchel and Füchtenbush 1998) – such transformation was recognized as a very proficient survival strategy used by several microorganisms (Babel et al. 2001). The biopolyesters resemble to plastics or elastomers derived from petroleum depending on their chemical structure (Steinbüchel and Füchtenbush 1998), although PHAs can be completely metabolized to CO_2_ and water under aerobic conditions or to methane and CO_2_ in anaerobic environments by various organisms.

Microorganisms transform sugars and fatty acids to PHAs through metabolic pathways that involve as intermediate either acetyl-CoA or acyl-CoA and conclude with monomer polymerization by PHA synthases (Philip et al. 2007). The ability of microorganisms to synthesize a particular form of PHA is mainly due to the substrate specificity of PHA synthases; these enzymes may be divided into four classes (Rehm 2003). PHA synthases belonging to class I utilize CoA thioesters of 3-hydroxyalkanoates (3-HAs) comprising 3–5 carbon atoms, whereas class II polymerases direct their specificity toward CoA thioesters of 3-HAs with 6–14 carbon atoms. Synthases of both classes I and II are encoded by PhaC gene. Class III synthases are composed of two genes (PhaC and PhaE) that possess substrate specificities similar to class I, although the PhaCE subunit can also polymerize 3-HAs with 6–8 carbon atoms. Class IV synthases are also composed of two genes (PhaC and PhaR) that utilize 3-HA monomers with 3–5 carbon atoms (Rehm 2003). Poly(3-hydroxybutyrate) (PHB) is the most common type of the PHAs synthesized by microorganisms, and is rigid and brittle (Steinbüchel and Füchtenbush 1998; Philip et al. 2007). However, copolymers with varying monomer compositions can also be produced resulting in a high diversity of PHA molecules possessing a broad range of physico-chemical and mechanical properties, for example, poly(3-hydroxybutyrate-*co*-hydroxyvalerate) (PHBV) that is a more flexible material than PHB (Steinbüchel and Füchtenbush 1998; Philip et al. 2007). PHAs are also biocompatible and lack toxicity (Philip et al. 2007). Owning to this features, PHAs have been used to develop some devices for medical applications including biodegradable sutures, meniscus repair devices, bone plates, heart valves, nerve conduits, and drug delivery systems (Chen and Wu 2005; Wu et al. 2009).

Studies on production of PHAs by halophilic, salt (NaCl) requiring microorganisms, were recently initiated (Quillaguamán et al. 2010). The advantage of using of halophilic microorganisms in PHA production systems is related to their ability to grow optimally at high salt concentrations (Quillaguamán et al. 2010). At determined concentrations of salt, the growth of nonhalophilic microorganisms is prevented, hence allowing a process without strict sterile conditions and reducing the inherent costs, such as the costs of the energy required for sterilizing the equipment for fermentation and culture media (Quillaguamán et al. 2010). Nevertheless, salts in the medium are to be concentrated and recycled in order to reduce the overall process costs as well as to minimize ecological pollution implicit in the disposal of the fermentation residues (Quillaguamán et al. 2010). Several halophilic archaea and bacteria isolated from marine-related niches are able to accumulate PHA, albeit only a few reached yields and volumetric productivities high enough to be considered for industrial purposes (Quillaguamán et al. 2010). However, the biotechnological potential of halophiles remains to be studied further. Mangrove forests in Northern Vietnam are saline coastal habitats composed by shrubs and medium height trees. Mangrove ecosystems are rich in organic matter; however, they are usually nutrient-deficient, especially in nitrogen and phosphorus (Alongi et al. 1989). Many different microorganisms including bacteria, fungi, protozoa, and algae have been found in mangrove ecosystems (Holguin et al. 2001). Among these microbes, the bacterial population is many-fold greater than the others. Because of its diversity, bacterial activity is responsible for most of the mineral cycle and the carbon flux in the mangrove ecosystems, and act as a carbon sink (Holguin et al. 2001).

This article reports the production of PHAs by halophilic and halotolerant bacterial species isolated from mangrove soil samples in Northern Vietnam – these environments have not been microbiologically studied. The isolates were identified by molecular analysis of their 16S rDNA sequences and phenotypic characterization. The chemical structure of the polymer synthesized by these microorganisms was determined by nuclear magnetic resonance (NMR) spectroscopy analysis. The polymer inclusions, their size, number, and organization in the cells were studied under electron microscopy. Furthermore, the cell densities and polymer yields reached by the isolates were also evaluated using either glucose or a mixture of glucose and propionate in the culture medium.

## Materials and Methods

### Isolation of bacterial strains

Soil samples from mangroves in Northern Vietnam at Giao Thuy district, Nam Dinh province, and at Yen Hung district, Quang Ninh province, were collected and serially diluted with sterile sea water, and then 100 μL of the dilution was spread on solid HM medium (Quillaguamán et al. 2004), containing (g/L): NaCl, 30; MgSO_4_·7H_2_O, 0.25; CaCl_2_, 0.09; KCl, 0.5; NaBr, 0.06; peptone, 5.0; yeast extract, 10.0; glucose, 1.0; and granulated agar, 20; and pH adjusted to 7 using 2 N NaOH. The plates were incubated at 35°C for 30 h. Several hundreds of colonies were isolated by plating them again on fresh agar medium.

### Detection of PHA in bacteria

Bacterial isolates were grown on a modified solid HM medium (HM-1) containing (g/L): NaCl, 30; MgSO_4_·7H_2_O, 0.25; CaCl_2_, 0.09; KCl, 0.5; NaBr, 0.06; KH_2_PO_4_, 0.25, yeast extract, 2.0; glucose, 20; granulated agar, 20; and Nile red (Sigma, Steinheim, Germany) (dissolved in dimethylsulfoxide) with a final concentration of 0.5 μg dye per mL of the medium (Spiekermann et al. 1999). Petri dishes were incubated at 35°C for 2 days. The agar plates were then exposed to untraviolet light (312 nm) to detect the presence of intracellular PHA granules in the bacteria (Spiekermann et al. 1999). The colonies with fluorescent bright orange were chosen for further studies.

### Transmission electron microscopy observation

PHB-containing cells were fixed and observed under transmission electron microscopy (TEM) following a protocol reported previously (Quillaguamán et al. 2006). Cells were separated by centrifugation at 4000*g* for 7 min and fixed for 4 h at room temperature in a solution of 4% (v/v) glutaraldehyde in 0.1 mol/L sodium cacodylate, pH 7.1, and 0.1% (w/v) Brij 35, followed by an overnight treatment in the same solution without Brij 35. The cells were then rinsed with 0.1 mol/L sodium cacodylate, pH 7.1, transferred to 2% osmium tetroxide for 8 h at room temperature and subsequently to 2% uranyl acetate in 10% ethanol for 40 min. The cells were dehydrated through a graded series of ethanol–water solutions with a final treatment in propylene oxide, and embedded in epon/araldite resin that was then cut with a diamond knife. The fine sections of 50 nm were placed on Formvar-coated copper grids, contrasted with a 2% aqueous solution of uranyl acetate and examined under a JEM-1010 transmission electron microscope (Jeol Korea Ltd., Korea).

### Phylogenetic and phenotypic characterization of the selected PHA-accumulating bacteria

The morphological and physiological properties of the selected PHA-accumulating bacteria were investigated according to Bergey's Manual of Determinative Bacteriology. For phylogenetic studies, the 16S rDNA of the bacteria was amplified by PCR using universal primers: 314F (5′-CCTACGGGAGGCAGCAG-3′) and 907R (5′-CCGTCAATTCCTTTGAGTTT-3′), and 27F (5′-AGAGTTTGATCCTGGCTCAG-3′) and 1492R (5′-GGTTACCTTGTTACGACTT-3′). Sequencing of the amplified DNA fragment was performed at Bioneer, Korea. GenBank and Ribosomal Database Project databases were used to seek for 16S rDNA gene similarities. Phylogenetic analysis based on the 16S rDNA gene was performed with the aid of the Mega 5 software package (Tamura et al. 2011), using the neighbor-joining distance correction methods (Saitou and Nei 1987). For constructing a phylogenetic tree, only sequences from the type strains of species whose names have been validly published were taken into account. Almost complete sequences (c.a. 1400 bp) of the 16S rDNA genes of the strains isolated in Vietnam were deposited at GenBank/EMBL/DDBJ databases and were used in the analysis.

### Production of PHA by the isolated strains

The selected bacterial strains were grown in 20 mL of HM medium in 100 mL flasks at optimum NaCl concentration and temperature for each strain with rotary shaking at 180 rpm for 13 h. Subsequently, 2.5 mL of each culture was inoculated in 250 mL Erlenmeyer flasks containing 50 mL of HM-1 medium with optimum NaCl concentration for each strain. The pH of this medium was initially adjusted to 7.0 using 5 mol/L NaOH. The cultures were incubated at 32°C for Gram-negative bacteria and at 37°C for Gram-positive bacteria with rotary shaking at 180 rpm. In all cases, samples were withdrawn at 30 h of cultivation for cell dry weight (CDW) determination and PHA content analysis.

### Quantitative analysis

CDW was determined by centrifuging 3 mL of the culture samples at 4000 *g* for 10 min in a preweighed centrifuge tubes, the pellet was washed once with 3 mL distilled water, centrifuged and dried at 105°C until constant weight was obtained. The centrifuge tube was weighed again to calculate the CDW.

PHA content analysis was performed using a gas-chromatographic method (Huijberts et al. 1994). For this, about 10 mg of freeze-dried cells was mixed with 1 mL of chloroform and 1 mL of methanol solution containing 15% (v/v) sulfuric acid and 0.4% (w/v) benzoic acid. The mixture was incubated at 100°C for 3 h to convert the constituents to their methyl esters. After cooling to room temperature, 0.5 mL of distilled water was added and the mixture was shaken for 30 sec. The lower chloroform layer was transferred into a fresh tube and used for GC analysis to determine the PHA content. Sample volume of 2 μL was injected into the gas chromatography column (VARIAN, Factor Four Capillary Column, CP8907). The injection temperature was 250°C, the detector temperature was 240°C, and the column temperature was 60°C for the first 5 min and then increased at 3°C/min until 120°C was reached. PHB and PHBV containing 12% valerate (Sigma) were used as a standard for calibration.

### PHA isolation for NMR spectroscopic analysis

NMR analysis was performed as described previously (Quillaguamán et al. 2006). The selected bacterial cells containing the polymer were harvested from 300 mL of culture broth by centrifugation at 6000 *g* for 10 min, washed twice with distilled water and lyophilized. PHA was recovered from lyophilized cells by extraction for 30 h with chloroform in a Soxhlet apparatus, and concentrated by evaporating the solvent under vacuum. The polymer was precipitated from the concentrated solution with 10 volumes of ethanol and the resulting PHA granulates were filtered twice. The ^1^H NMR spectrum was recorded at 500 MHz with a Bruker ARX500 Spectrometer (Bruker, Sikerstrifen, Germany) at room temperature using deuterated chloroform as internal reference solvent. The spectrum was evaluated using standard Bruker UXNMR software.

## Results

### Isolation and screening of PHA-accumulating bacteria

**Figure 1:**
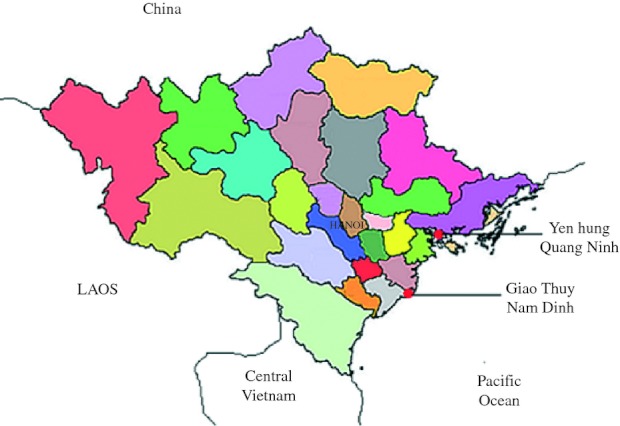
Regions in Vietnam where mangrove forests are located. Mangrove forests located at Giao Thuy district in Nam Dinh province and Yen Hung district in Quang Ninh province were selected to isolate halophilic and halotolerant microorganisms.

Soil samples collected from mangrove forests at Giao Thuy district, Nam Dinh province, and at Yen Hung district, Quang Ninh province in Northern Vietnam (Fig. 1), were used for this study. Mangrove forests are specialized ecosystems situated at the interphase between land and sea of tropical and subtropical areas (Spalding et al. 2010). We hypothesized that the presence of excess carbon and limitation of nitrogen and phosphorus in mangrove forests (Spalding et al. 2010) could be a favorable condition for the existence of microbes that have the ability to accumulate PHA.

The soil samples of the forests were inoculated on solid HM medium, which was previously used to isolate halophilic microorganisms (Quillaguamán et al. 2004). After 30 h of incubation at 35°C, about 500 bacterial colonies were obtained on agar plates. Bacterial colonies were stained with Nile Red dye and PHA-producing bacteria were identified by examining them in a fluorimeter. About 100 fluorescent bacteria were observed to accumulate PHAs; among them, eight bacterial strains that exhibited a very strong fluorescence were selected to be studied further. The strains were named as ND97, ND153, ND199, ND218, ND240 (ND standing for the strains isolated from Nam Dinh), and QN187, QN194, QN271 (QN standing for the strains isolated from Quang Ninh).

### Phylogenetic studies based on 16S rDNA sequences

**Figure 2:**
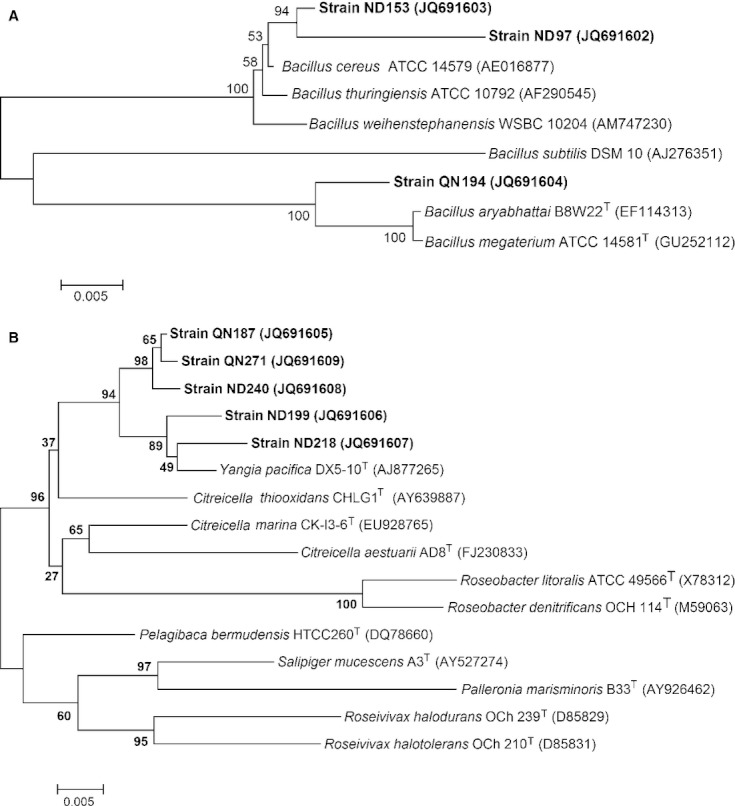
Phylogenetic trees constructed using 16S rDNA gene sequences of (A) Gram-positive bacteria belonging to the genus *Bacillus* and (B) Gram-negative bacteria within the α-Proteobacteria. Bar, five substitutions per 1000 nucleotides. Numbers at branching points refer to bootstrap values (500 resamplings).

The phylogenetic affiliation of the eight bacterial strains selected was analyzed using their 16S rDNA sequences. The sequences of strains ND153, ND97, and QN194 share a close relationship with sequences of *Bacillus* species (Fig. 2A). Strains ND153 and ND97 clustered together and had a 16S rDNA similarity of 98.3%. The closest similarity of strains ND153 and ND97 was shared with *Bacillus cereus*, 99.3% and 98%, respectively, whereas the 16S rDNA sequence of strain QN194 was 98.2% similar to the sequences of *B. aryabhattai* and *B. megaterium* (Fig. 2A).

On the other hand, strains QN187, ND199, ND218, ND240, and QN271 gathered with the α-proteobacterium *Yangia pacifica* (Fig. 2B). The 16S rDNA sequences of these strains were 98.4–98.9% similar to *Y. pacifica*. Other α-proteobacteria that belong to the genera *Citreicella* and *Roseivivax* were found in different clades, and sheared a similarity of 97.4% or lower with the strains isolated from Vietnam.

### Phenotypic characterization of the selected bacterial strains isolated from mangroves

The morphological and physiological characteristics of the eight selected strains are summarized in Table 1. All strains were aerobic, rod-shaped, and motile. They were also catalase and oxidase positive and showed a negative test for indol formation (Table 1). All strains were mesophilic with optimum temperatures for growth varying from 30 to 40°C and were able to grow with an optimum pH between 6 and 7 (Table 1). Three strains (ND97, ND153, and QN194) were Gram positive, spore forming, and halotolerant (optimum growth between 0% and 1% NaCl), the remaining five strains (ND199, ND218, ND240, QN187, and QN271) were Gram negative, nonspore forming, and halophilic bacteria (optimum growth between 3% and 7% NaCl) (Table 1). Moreover, the Gram-positive strains were gelatinase, caseinase, and amylase positive and were urease negative, which differ from the tests for Gram-negative strains (Table 1). Regarding the assimilation of carbon sources, l-arabitol, carboxy methyl cellulose, α-methyl-d-mannose, α-methyl-d-glucose were not suitable substrates for the growth of the eight strains, whereas d-raffinose, maltodextrin, maltose, fructose, inulin, glucose, sucrose, cellobiose, and cane molasses promoted the growth of all strains (Table 1).

**Table 1:** Phenotypic characteristics of the bacteria isolated from soil at mangrove forests in Vietnam and the reference strains *Bacillus cereus* and *Yangia pacifica*

	ND97	ND153	QN194	*B. cereus*	QN187	ND199	ND218	ND240	QN271	*Y. pacifica*
Morphological characteristics										
Shape	Rod	Rod	Rod	Rod	Rod	Rod	Rod	Rod	Rod	Rod
Size (μm)	0.7–1.2 × 1.6–3.0	0.8–1.2 × 2.0–3.5	0.5–0.7 × 1.0–2.5	>0.9 × >3.0	0.4–0.7 × 1.5–3.0	0.8–1.1 × 1.2–2.2	0.4–0.6 × 1.0–3.0	0.3–0.5 × 1.5–3.5	0.4–0.7 × 1.4–2.5	0.8 × 1.0–1.5
Motility	+	+	+	+	+	+	+	+	+	+
Gram staining	+	+	+	+	−	−	−	−	−	−
Spore formation	+	+	+	+	−	−	−	−	−	−
Growth conditions										
Optimum temperature (°C)	35–37	35–37	37–40	37	30–33	30–33	33–35	30–33	33–35	37
Optimum pH	6–7	6–7	6–7	6–8	6.5–7.5	6.5–7.5	7–8	7–8	6.5–7.5	7.5
Optimum NaCl (%, w/v)	0–1.0	0–1.0	0–1.0	<2.0	2–3	4–5	6–7	4–5	4–5	5
Aerobic conditions	+	+	+	±	+	+	+	+	+	+
Physiological characteristics										
Catalase	+	+	+	+	+	+	+	+	+	+
Oxidase	+	+	+	+	+	+	+	+	+	±
Urease	−	−	−	−	+	+	+	+	+	+
Gelatinase	+	+	+	+	−	−	−	−	−	+
Caseinase	+	+	+	+	−	−	−	−	−	+
Amylase	+	+	+	+	−	−	−	−	−	+
Indol formation	−	−	−	−	−	−	−	−	−	−
Methyl red test	+	+	−	+	−	−	−	−	−	−
Voges–Proskauer test	+	+	−	+	−	−	−	−	−	−
Growth on										
Citrate	−	−	+	+	+	+	+	+	+	NR
d-Galactose	−	−	+	−	+	+	+	+	+	NR
d-Gluconate	−	−	+	−	+	+	+	+	+	NR
d-Fucose	−	−	−	NR	+	+	+	+	+	NR
l-Fucose	−	−	−	NR	+	+	+	+	+	NR
l-Arabitol	−	−	−	NR	−	−	−	−	−	NR
Starch	+	+	+	+	−	−	−	−	−	+
d-Raffinose	+	+	+	−	+	+	+	+	+	NR
Maltodextrin	+	+	+	NR	+	+	+	+	+	NR
Maltose	+	+	+	+	+	+	+	+	+	+
Mannitol	−	−	+	−	+	+	+	+	+	−
Lactose	−	−	+	−	+	+	+	+	+	−
Fructose	+	+	+	+	+	+	+	+	+	−
l-Rhamnose	−	−	−	−	+	+	+	+	+	NR
d-Xylose	−	−	+	−	+	+	+	+	+	NR
Inositol	+	+	+	−	+	−	−	+	+	−
Sorbitol	−	−	+	−	+	+	+	+	+	−
Inulin	+	+	+	NR	+	+	+	+	+	NR
Salicin	−	−	+	+	−	−	−	−	−	NR
Glucose	+	+	+	+	+	+	+	+	+	−
Sucrose	+	+	+	+	+	+	+	+	+	−
Carboxy methyl cellulose	−	−	−	NR	−	−	−	−	−	NR
Cellobiose	+	+	+	+	+	+	+	+	+	NR
α-Methyl-d-mannose	−	−	−	NR	−	−	−	−	−	NR
α-Methyl-d-glucose	−	−	−	NR	−	−	−	−	−	NR
Dextrin	+	+	+	NR	+	+	+	+	+	NR
Glycerol	−	−	+	+	+	+	+	+	+	NR
Cane molasses	+	+	+	NR	+	+	+	+	+	NR

Data for *B. cereus* were reported by Priest et al. (1988) and data for *Y. pacifica* were determined by Dai et al. (2006). +, positive; −, negative; NR, not reported.

Furthermore, the phenotypic characteristics were compared with those of *B. cereus*. Most characteristics of the Gram-positive strains and *B. cereus* were similar (Table 1); only some biochemical tests and growth on a few carbohydrates were different between the strains isolated from mangroves and *B. cereus* (Table 1). The phenotypic features of the Gram-negative strains QN187, ND199, ND218, ND240, and QN271 and those reported for *Y. pacifica* were also compared (Table 1). The Gram-negative strains and *Y. pacifica* differed with respect to the hydrolysis of casein, gelatine, and starch (Table 1). They also differ regarding the growth on starch, mannitol, lactose, fructose, inositol, sorbitol, glucose, and sucrose (Table 1).

### Electron microscopy observation of microbial isolates containing PHA inclusions

**Figure 3:**
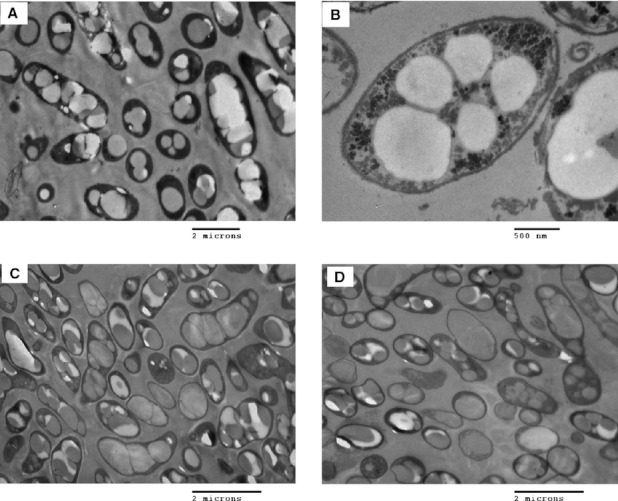
Transmission electron microscope pictures of (A) Strain ND153, (B) Strain ND97, (C) Strain QN187, and (D) Strain QN271 grown on HM-1 medium. The samples were taken after 30 h of cultivation.

The presence of PHA in the eight selected bacterial strains was analyzed using TEM. The cells with the highest contents of PHA were chosen for the micrographs (Fig. 3A–D). The Gram-positive strains ND253, ND97, and QN194 were rod cells with size ranging from 4.4 × 1.4 to 4.4 × 1.7 μm and coccid-shaped cells with an average size of 1.5 × 1.2 μm (Fig. 3A and B). Furthermore, these cells contained PHA inclusions with three different average diameters, c.a. 0.17, 0.27, and 1.2 μm. Coccoid cells contained one, two, or three PHA inclusions, while rods stored from four to nine PHA granules shifting in size (Fig. 3A and B). The Gram-negative strains QN187, ND199, ND218, ND240, and QN271 were short rods with sizes ranging from 2 × 0.3 to 2.3 × 1 μm and coccoid cells with an average diameter of 0.3 μm (Fig. 3C and D). The inclusions in Gram-negative bacteria had three average diameters, c.a. 0.18, 0.30, and 0.91 μm. The coccoid cells were completely filled with either one PHA inclusion or may store up to four inclusions (Fig. 3C and D). Moreover, short rods contained from 1 to 9 inclusions (Fig. 3C and D).

### PHA production by halophilic and halotolerant strains isolated from mangroves

**Figure 4:**
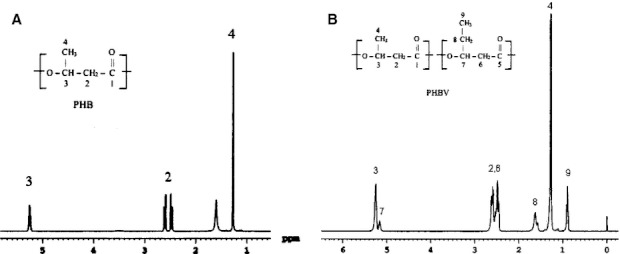
500 MHz ^1^H-NMR spectra of (A) purified PHB isolated from strain QN194 grown on glucose (2%, v/w) as carbon source, (B) purified PHBV isolated from strain ND153 grown on glucose (2%, v/w) and propionate (0.2%, v/w) as carbon sources.

The PHA produced by the eight selected strains was studied using either one or two sources of carbon. For a first set of experiments, an initial concentration of 20 g/L glucose was used as sole carbon source. For a second set of experiments, propionate was also added to the culture medium to reach an initial concentration of 0.2 g/L after 10 h of cultivation. CDW, PHA content, and the polymer composition were evaluated in all assays (Table 2). For the first group of experiments, we determined that strains ND199 and ND218 were able to synthesize the copolymer PHBV from glucose while the remaining strains accumulated PHB. Table 2 shows also that strain ND153 accumulated the largest amount of PHA (65 wt%). Under the same culture conditions, strains ND97, QN187, and QN271 stored the polymer in yields between 44 and 48 wt%. The remaining strains attained yields lower than 35 w% (Table 2). The addition of propionate in the culture medium enhanced the final yield of PHA reached by strains ND97 and ND153, and induced the synthesis of PHBV in seven of the eight strains (Table 2). The cell growth, as determined by the CDW, reached by seven of the strains was between 2 and 3.8 g/L while strain QN271 achieved a slightly higher CDW, c.a. 4.7–5.1 g/L (Table 2). The chemical structures of the PHAs were confirmed further by ^1^H-NMR analysis (Fig. 4). The proton signals and chemical shifts clearly showed that two different types of PHAs were synthesized by the strains, that is, PHB (Fig. 4A) and PHBV (Fig. 4B).

**Table 2:** Cell growth, PHA content, and composition attained by halophilic and halotolerant strains isolated from soil at mangrove forests in Vietnam

	Carbon substrate							
	
	Glucose				Glucose + Propionate^1^			
		
			PHA composition				PHA composition	
						
Strain	CDW (g/L)	PHA content (wt%)	3HB (mol%)	3HV (mol%)	CDW (g/L)	PHA content (wt%)	3HB (mol%)	3HV (mol%)
ND97	3.1	48	100	0	3.3	53	86	14
ND153	3.1	65	100	0	3.6	71	91	15
QN194	2.2	26	100	0	1.8	11	100	0
ND199	2.6	34	98	2	2.1	12	56	44
ND218	2.3	24	97	3	2	11	79	21
QN271	5.1	48	100	0	4.7	31	95	5
QN187	3.8	44	100	0	3.1	27	90	10
ND240	3.2	28	100	0	2.6	12	87	13

All experiments were performed in shake flasks.

1 A propionate concentration of 0.2 g/L was fed into the culture after 10 h of growth.

## Discussion

Mangrove forests are characterized for presenting a much higher content of organic matter than nitrogen or phosphorous sources (Alongi et al. 1989). Moreover mangrove forests in Vietnam are coastal areas, whereby they are constantly in contact with salts coming from sea; bacterial diversity in mangroves is known to be responsible of most of the carbon cycle (Holguin et al. 2001). Bacteria that succeeded in such ecosystems should have adapted their metabolisms to variations in salt concentrations, excess in carbon sources, and limited amounts of other essential nutrients such as nitrogen or phosphorous. Several halophilic and halotolerant bacteria are able to tolerate a wide range of NaCl concentrations (Oren 2008), whereas various species accumulate polyesters (Quillaguamán et al. 2010). In this regard, we sought for halophilic or halotolerant microorganisms in Vietnamese mangrove forests that were able to synthesize PHAs in large amounts.

We obtained about 100 halophilic and halotolerant isolates able to produce PHAs from mangrove forests located at the provinces of Nam Dinh and Quang Ninh in Vietnam. Therefore, synthesis of PHA shows to be a strategy of adaptation that microorganisms follow in such ecosystems, and might have a role to play in the carbon cycle in the mangrove forests. Three of the eight selected strains belonged to the genus *Bacillus* (Fig. 2A). Different *Bacillus* species share 16S rDNA similarities above 99% and exhibit only a few phenotypic differences, but DNA relatedness among them revealed that they are genetically distinct species (Priest et al. 1988; Nakamura and Jackson 1995). Consequently, DNA–DNA hybridization studies may help to discern the species association of strains ND97, ND153, and QN194. Members of the genus *Bacillus* are highly ubiquitous in nature. (Priest et al. 1988; Nakamura and Jackson 1995; Lechner et al. 1998). Strains of *B. cereus*, *B. thuringiensis*, *B. megaterium*, *B. aryabhattai,* and other bacilli that form part of their phylogenetic cluster were also found in marine-related ecosystems (Ettoumi et al. 2009; Jung et al. 2011; Antony et al. 2012). Moreover, strains of *B. amyloliquefaciens* and *B. megaterium* were isolated in mangrove forests; the former possessed larvicidal activity and the latter was able to reduce selenite (Geetha et al. 2011; Mishra et al. 2011). In this sense, strains ND97, ND153, and QN194 could also interact with other organisms at the coastal areas that they habit, besides their capacity to synthesize polyesters.

The first studies on the production of PHB by microorganisms were performed on *B. megaterium* (Lemoigne 1926). Several other *Bacillus* species including *B. thuringiensis* and *B. cereus* were also found to be able to store PHB (Kominek and Halvorson 1965; Chen et al. 1991). *Bacillus megaterium*, *B. thuringiensis*, and *B. cereus* include genes that encode class IV PHA synthases in their genomes (Tseng et al. 2006; Hyakutake et al. 2011). Class IV synthases polymerize PHAs containing short-chain-length monomers such as PHB and PHBV (Rehm 2003). Strains ND97, ND153, and QN194 cells had one or three PHA inclusions, while rods stored from four to nine PHA granules varying in size (Fig. 3A and B). A similar number of PHA inclusions were found in *B. megaterium* (McCool et al. 1996). Strain ND153 accumulated large amounts of PHA, 65–71 wt% (Table 2), which are higher than the largest obtained by most *Bacillus* species, with yields ranging between 40 and 47 wt% (Valappil et al. 2007). It is also noteworthy that strain ND153 assimilates sucrose and sugarcane molasses (Table 1). These substrates are cheaper alternatives than glucose for the production of polyesters by *Bacillus* species (Valappil et al. 2007; Kumar et al. 2009; Akaraonye et al. 2012). The use of cheap substrates leads to feasible bioprocesses, which also become environmentally friendly when agricultural surplus such as molasses are used as the source for polyester production.

Gram-negative strains isolated from Vietnamese mangrove forests, that is, strains QN187, ND199, ND218, ND240, and QN271, were phylogenetically gathered with *Y. pacifica* (Fig. 2B). However, these strains showed various biochemical and nutritional differences with *Y. pacifica* (Table 1), implying that they are different strains from the type strain of *Y. pacifica*. Additional DNA relatedness studies are required to establish whether these strains belong to the *Y. pacifica* species. On the other hand, there are no reports on the production of PHA by *Y. pacifica*, albeit the strains isolated from Vietnam were able to produce PHA (Fig. 3C and D Table 2). The number and size of PHA granules accumulated by Gram-negative bacteria fluctuate depending on the phylogenetic group. The β-proteobacterium *Cupriavidus necator* (formerly called *Ralstonia eutropha*) cells store between 8 and 12 PHB granules with varying diameters in the range of 0.24–0.50 μm (Anderson and Dawes 1990), whereas the γ-proteobacterium *Azotobacter vinelandii* can accumulate more than 40 granules per cell, with sizes of 0.5–1.4 μm (Page et al. 1995). Moreover, the halophilic γ-proteobacterium *Halomonas boliviensis* commonly synthesizes one or two granules (0.20–0.64 μm) per cell, although occasionally the formation of up to five granules in elongated cells was observed (Quillaguamán et al. 2006). The organization, size, and number of inclusions found in the strains isolated from Vietnam (Fig. 3C and D) are similar to those found in *H. boliviensis*. The formation of such large and uniform PHB granules is suggested to be advantageous for the purification and quality of the polymer (Steinbüchel et al. 1995).

Interestingly, two of the Gram-negative strains, that is, ND199 and ND218 (Table 2), could synthesize the copolymer PHBV using glucose as carbon source. Only a few bacteria such as *Rhodococcus* species (Valentin and Dennis 1996) and halophilic archaea (Quillaguamán et al. 2010) are able to synthesize PHBV without the inclusion of propionic or valeric acid in the microbial culture medium. The halophilic archaea able to accumulate PHBV from carbohydrates possess PHA synthases that belong to the class III (Quillaguamán et al. 2010). Molecular analysis supports that the genes encoding PHA synthases of class III were transferred between bacteria and archaea able to thrive in marine-related ecosystems (Quillaguamán et al. 2010). Nevertheless, halophilic *Halomonas* species harbor genes that encode synthases phylogenetically close related to class I (Quillaguamán et al. 2010; Guzmán et al. 2012). The closest identities of the enzymes found in halophilic bacteria are shared with Proteobacteria of different subgroups, that is, α, β, and γ (Quillaguamán et al. 2010; Guzmán et al. 2012). Both class I and class III PHA synthases direct the production of PHBV when a carbohydrate and propionate form part of the production medium of the microorganisms (Rehm 2003); therefore, only additional molecular studies will reveal the type of PHA synthases that are expressed by the strains isolated from Vietnam. The maximum PHB yield and CDW reached in batch systems by strain QN271 were 48 wt% and 5.1 g/L, respectively (Table 2), which are rather lower than those reached by *H. boliviensis* (54 wt% and 14 g/L) (Quillaguamán et al. 2007), *Cupriavidus necator* (54 wt% and 9.4 g/L) (Doi et al. 1988), and a recombinant *Escherichia coli* strain (80.8 wt% and 8.9 g/L) (Lee et al. 1994). These bacteria attained among the highest productions of PHB and are recognized for their potential and current utilization at industrial scales. Studies on the optimization of the culture medium of strain QN271 that lead to higher polymer yields and cell growth using combinations of carbon sources are in progress. The studies should discern the potential that strain QN271 has for large-scale production systems.
